# Hook plate for medial clavicle fracture

**DOI:** 10.4103/0019-5413.61768

**Published:** 2010

**Authors:** J Gille, AP Schulz, S Wallstabe, A Unger, C Voigt, M Faschingbauer

**Affiliations:** Department of Trauma and Reconstructive Surgery, BG- Trauma Hospital Hamburg, Bergedorfer Str. 10, 21033 Hamburg, Germany; 1 Friederikenstift GmbH Hannover, Humboldstr. 5, 30169 Hannover, Germany

**Keywords:** Hook plate, medial clavicle fracture, sternoclavicular dislocation

## Abstract

Medial clavicle fractures are the least common type of clavicular fractures. Although rare, such injuries deserve rapid diagnosis and effective treatment to avoid future complications. An optimal, standardized operative treatment has not been yet established. We report a case of medial clavicle fracture, where primary operative treatment was indicated due to gross dislocation. An open reduction and osteosynthesis with a modified hook plate was performed, leading to an excellent postoperative outcome after a sixteen-month follow-up. The hook plate seems to be a promising approach for the operative treatment of medial clavicle fractures.

## INTRODUCTION

The articulation of the clavicle medially with the manubrium of the sternum forms the sternoclavicular joint and represents the only articular link to the axial skeleton, the remainder of the pectoral girdle being suspended by the muscles. Clavicle fractures are common, representing around three per cent of all fractures.^1^ Fractures to the medial end of the clavicle are uncommon, representing only two to four per cent of all clavicle fractures.^2^ However, they are typically accompanied by significant multisystem trauma and have a high associated mortality rate.^3^ According to the direction of clavicle fracture dislocation, two types are described – anterior and posterior dislocation. In anterior dislocation, the fractured clavicle dislocates ventrally; in a posterior dislocation dorsally, in relation to the sternum.^4^ Posterior dislocation may give rise to serious complications such as compression and tear of the adjacent neural and vascular structures, the trachea and esophagus and the occurrence of pneumothorax.^5^

Although rare, such injuries deserve rapid diagnosis and effective treatment to avoid any future complication. Conservative or operative treatment protocols^2^,^3^ for sternoclavicular fracture have been established in the past. Due to the proximity of important structures they have often been treated conservatively to avoid intraoperative complications. An optimal, standardized operative treatment has not been yet established because of the small number of cases. We describe a technique of using a modified hook plate to fix a displaced medial clavicle fracture as one of the treatment options.

## CASE REPORT

**Figure 1:**
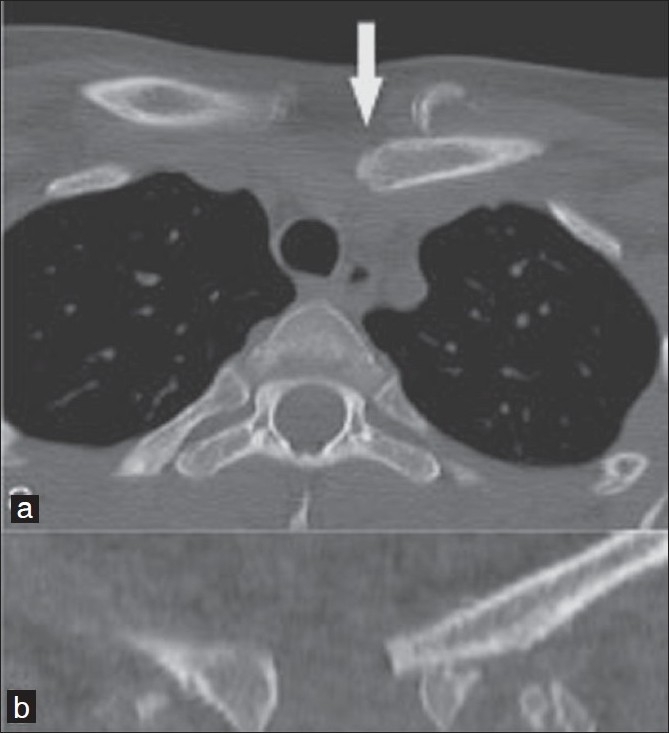
(a,b) Axial CT at the clavicular level showing a medial and posterior displacement of the medial end of the left clavicle (solid arrow) and revealing a medial fracture of the clavicle

We report the case of a 21-year-old female patient who was involved in a road traffic accident. She complained about a feeling of pressure at the inlet of the thorax and a local pressure pain projecting towards the sternoclavicular joint on the left side. Clinical examination indicated a malalignment of the left sternoclavicular joint when compared to the contralateral side with tenderness to pressure and percussion. There was no open injury, dysphagia, dyspnea or numbness of the left upper extremity and the blood pressure was normal. Radiological examination indicated a dislocation of the sternoclavicular joint. Axial CT at the clavicular level demonstrated a medial and posterior displacement of the medial end of the left clavicle and revealed a medial fracture of the clavicle [Figure 1a and b]; plain X-ray films did not pinpoint to the fracture.

**Figure 2:**
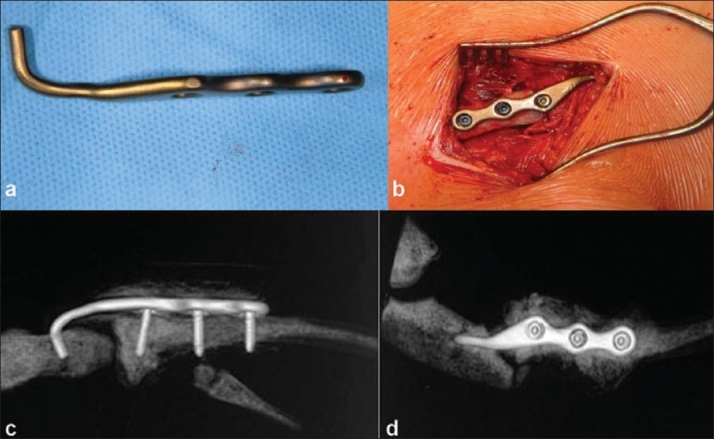
(a) Hook plate (Litos, Hamburg, Germany) after modification to suit the anatomical situation at the sternoclavicular joint; (b) intraoperative photograph showing repositioning of hook into the burred hole in sternum and the plate fixation with three cortical screws. (c) and (d) The postoperative CT showing a perfect reduction of the medial clavicle and the sternoclavicular joint

Due to gross dislocation of the fracture, no attempt of closed reduction was made. Under general anesthesia, the patient, positioned in decubitus position with the dorsum inclined at around 30°, a transverse incision was made above the joint. After accessing the joint, subperiosteal dissection was performed while protecting the deep vascular structures. The fracture was exposed, repositioned and temporarily fixed. After modification of the hook plate [Figure 2a] to suit the anatomical landmarks the suitable hole was made in the sternum with a drill. After positioning of the hook the plate was fixed with screws in the clavicle [Figure 2b]. Postoperatively, the left upper extremity was immobilized in a Gilchrist sling for two weeks.^6^ Subsequently, the patient began early functional exercises. The postoperative computed tomographic (CT) assessment revealed a perfect reduction of the medial clavicle and the sternoclavicular joint [Figure 2c and d].

Implant removal was performed on an outpatient basis, 12 weeks postoperatively, without any complications. At six months follow-up examination, the patient was already in training for competitive handball. After 16 months follow up, clinically stability was present at the sternoclavicular joint and there was absence of pain.

## DISCUSSION

Fractures of the medial end of the clavicle are uncommon.^2^ Although rare, such injuries deserve rapid diagnosis and effective treatment to avoid future complications. They are often associated with polytrauma and therefore have a high associated mortality.^3^

Clinical examination often indicates a malalignment of the sternoclavicular joint when compared to the contralateral side with tenderness to pressure and percussion. Plain radiography may be diagnostic, showing an asymmetry of the sternoclavicular joint. Some authors have advocated additional views or even conventional tomography.^7^ It is now generally agreed that CT is the examination of choice, not only to precisely define the type of fracture and dislocation but also to reveal any associated injury of adjacent structures.^8^ In our case, CT scan elucidated the combination of a medial clavicle fracture with posterior fracture dislocation.

The recommendations for treating medial clavicle fractures ranges from conservative treatment to surgical reconstruction.^2^,^3^,^9^

Conservative treatment includes manual reduction, correcting tape, plaster and slings, symptomatic treatment of pain, local application of ice, and immobilization of the affected upper extremity for several weeks.^4^ Conservative treatment is often associated with cosmetic asymmetry, chronic pain, periarticular calcifications with ankylosis and progressive deformity.^1^ Surgical treatment is chosen on the one hand when closed reduction is not successful and on the other hand when the type of injury progresses to recurrent instability and pain, thereby causing great discomfort and incapacity.^10^ As mentioned above, an optimal, standardized operative treatment has not been yet established. The applied procedures include wire osteosynthesis, pin fixation with resorbable materials, complex capsular ligament reconstructions with displacement of tendons, resection of the medial end of the clavicle and arthrodesis of the sternoclavicular joint.^11^ When inserting transfixing resorbable or nonresorbable pins, there is a risk of implant failure and pin migration into the mediastinum, ventricular penetration and cardiac tamponade.^12^ For cosmetic reasons, resection of the medial end of the clavicle cannot be recommended despite good functional results. Biological reconstruction in sternoclavicular joint dislocation can be performed with autologous grafts, e.g. semitendinosus graft.^10^ Armstrong *et al*, report a new method using the tendon of the sternocleidomastoid muscle; the results were satisfactory in seven cases.^9^ In our case no biological reconstruction was possible because of the accompanying fracture of the clavicle.

After reduction of the fractured sternoclavicular joint, we used a hook plate, inspired by excellent results following temporary arthrodesis with the hook plate in acromioclavicular dislocation.^13^ As mentioned above, the operative procedure was successful without any complication. The clinical results of the presented case encourage us to use the hook plate in the future regularly in medial clavicular fractures and complex sternoclavicular injuries. There is no doubt that the hook plate may cause complications that are inherent to all nonbiological implants. Implant removal is recommended; thus a second operation is necessary and may account as a disadvantage of the procedure. Sixteen months after the operation, the patient is symptom-free and no further dislocation has occurred. The hook plate is a promising approach for the operative treatment of medial clavicle fractures.

One weakness of this study is that conclusions are only based on one case, always hardly sufficient to make a generalized conclusion. However, additional studies using the case-study method may elucidate the truth of the conclusion drawn.

## References

[1] Nowak J, Holgersson M, Larsson S (2005). Sequelae from clavicular fractures are common: A prospective study of 222 patients. Acta Orthop.

[2] Postacchini F, Gumina S, de Santis P, Albo F (2002). Epidemiology of clavicle fractures. J Shoulder Elbow Surg.

[3] Throckmorton T, Kuhn JE (2007). Fractures of the medial end of the clavicle. J Shoulder Elbow Surg.

[4] Kälicke T, Westhoff J, Gekle Ch (2003). Anterior sternoclavicular dislocation caused by indirect compression trauma. Eur J Trauma.

[5] O'Connor PA, Nölke L, O'donnell A, Lingham KM (2003). Retrosternal dislocation of the clavicle associated with a traumatic pneumothorax. Interact Cardiovasc Thorac Surg.

[6] Lenza M, Belloti JC, Andriolo RB, Gomes Dos Santos JB, Faloppa F (2009). Conservative interventions for treating middle third clavicle fractures in adolescents and adults. Cochrane Database Syst Rev.

[7] Leighton D, Oudjhane K, Ben Mohammed H (1989). The sternoclavicular joint in trauma: Retrosternal dislocation versus epiphyseal fracture. Pediatr Radiol.

[8] Poelmann TA, Staal HM, Willems WJ (2008). A pseudo-iatrogenic case of medial clavicular fracture. Strategies Trauma Limb Reconstr.

[9] Armstrong AL, Dias JJ (2008). Reconstruction for instability of the sternoclavicular joint using the tendon of the sternocleidomastoid muscle. J Bone Joint Surg Br.

[10] Castropil W, Ramadan LB, Bitar AC, Schor B, de Oliveira D'Elia C (2008). Sternoclavicular dislocation--reconstruction with semitendinosus tendon autograft: A case report. Knee Surg Sports Traumatol Arthrosc.

[11] Bae DS, Kocher MS, Waters PM, Micheli LM, Griffey M, Dichtel L (2006). Chronic recurrent anterior sternoclavicular joint instability: Results of surgical management. J Pediatr Orthop.

[12] Gulcan O, Bolat B, Turkoz R (2005). Right ventricular penetration and cardiac tamponade as a late complication of Kirschner wire placement in the sternoclavicular joint. Interact Cardiovasc Thorac Surg.

[13] Habernek H, Schmid L, Walch G (1993). Management of acromioclavicular joint dislocation with the Wolter hook-plate: One year follow-up of 35 cases. Unfallchirurgie.

